# Genetic mechanism underlying sexual plasticity and its association with colour patterning in zebrafish (*Danio rerio)*

**DOI:** 10.1186/s12864-019-5722-1

**Published:** 2019-05-06

**Authors:** Shahrbanou Hosseini, Ngoc-Thuy Ha, Henner Simianer, Clemens Falker-Gieske, Bertram Brenig, Andre Franke, Gabriele Hörstgen-Schwark, Jens Tetens, Sebastian Herzog, Ahmad Reza Sharifi

**Affiliations:** 1 Department of Animal Sciences, University of Goettingen, Goettingen, Germany; 2 Center for Integrated Breeding Research, University of Goettingen, Goettingen, Germany; 3 Institute of Veterinary Medicine, University of Goettingen, Goettingen, Germany; 4 Institute of Clinical Molecular Biology, Christian-Albrechts-University, Kiel, Germany; 5 Max Planck Institute for Dynamics and Self-Organization, Goettingen, Germany; 6 Department for Computational Neuroscience, 3rd Physics Institute-Biophysics, University of Goettingen, Goettingen, Germany

**Keywords:** Zebrafish, Temperature, Embryogenesis, Sex ratio, Sex determination, Colour pattern, Masculinization, Transcriptome analysis

## Abstract

**Background:**

Elevated water temperature, as is expected through climate change, leads to masculinization in fish species with sexual plasticity, resulting in changes in population dynamics. These changes are one important ecological consequence, contributing to the risk of extinction in small and inbred fish populations under natural conditions, due to male-biased sex ratio. Here we investigated the effect of elevated water temperature during embryogenesis on sex ratio and sex-biased gene expression profiles between two different tissues, namely gonad and caudal fin of adult zebrafish males and females, to gain new insights into the molecular mechanisms underlying sex determination (SD) and colour patterning related to sexual attractiveness.

**Results:**

Our study demonstrated sex ratio imbalances with 25.5% more males under high-temperature condition, resulting from gonadal masculinization. The result of transcriptome analysis showed a significantly upregulated expression of male SD genes (e.g. *dmrt1, amh, cyp11c1* and *sept8b*) and downregulation of female SD genes (e.g. *zp2.1, vtg1, cyp19a1a* and *bmp15*) in male gonads compared to female gonads. Contrary to expectations, we found highly differential expression of colour pattern (CP) genes in the gonads, suggesting the ‘neofunctionalisation’ of those genes in the zebrafish reproduction system. However, in the caudal fin, no differential expression of CP genes was identified, suggesting the observed differences in colouration between males and females in adult fish may be due to post-transcriptional regulation of key enzymes involved in pigment synthesis and distribution.

**Conclusions:**

Our study demonstrates male-biased sex ratio under high temperature condition and support a polygenic SD (PSD) system in laboratory zebrafish. We identify a subset of pathways (tight junction, gap junction and apoptosis), enriched for SD and CP genes, which appear to be co-regulated in the same pathway, providing evidence for involvement of those genes in the regulation of phenotypic sexual dimorphism in zebrafish.

**Electronic supplementary material:**

The online version of this article (10.1186/s12864-019-5722-1) contains supplementary material, which is available to authorized users.

## Background

Mammals and avian species have a chromosomal sex determination (SD) mechanism in vertebrates with master switch of SD located on the sex chromosomes [1–4], whereas sex in teleost fish can be diverse and their sexual plasticity depends on genetic and environmental factors [5]. The genetic mechanism of SD in zebrafish (*Danio rerio*), a widely used model organism, is not fully understood, since there are no differences between the chromosomal sets of male and female genomes [2, 3]. In a previous study, it was found that a sex-associated region in zebrafish differs between wild and domesticated strains, located in the right telomere of chromosome 4 of wild populations. However, this sex-specific region was not found in domesticated strains [6], leading to the assumption that sex in domesticated strain is polygenetically determined [1, 3, 6, 7], in which the sex-determining genes are distributed over the whole genome [2]. Among a series of biotic and abiotic factors that influence the mechanism of SD in zebrafish during gonad development resulting in masculinization, temperature is the most important environmental factor [1, 8, 9]. During early embryonic development, the number of primordial germ cells (PGCs) plays an important role in gonad differentiation and sexual dimorphism [10, 11]. In order to form the primordial gonads, which later develop into a testis or an ovary, the PGCs form cell clusters and migrate during the first day of embryonic development toward the somatic cells of the gonad and merge with these cells to form germ cells. A subset of germ cells acquires the ability to operate as germ line stem cells, which later differentiate into gametes [10, 12]. During this critical embryonic developmental period, the loss or decrease in the number of PGCs may cause by increased water temperatures leads to masculinization [11, 13, 14]. This process is regulated in such a way that the testicular developmental genes are expressed and the expression of the ovarian developmental genes is inhibited in the “juvenile ovary” stage [8, 15]. The sex-reversed females are known as “neomales”, which possess testis and have similar gene expression profiles as normal males [1]. Hence, SD in zebrafish is controlled by the interaction between fish genotype and environmental factors (GxE) [1, 2, 13]. Elevated water temperature, e.g. caused by climate change, may induce male-biased populations, leading to an elevated risk of extinction in thermosensitive fish populations in nature [16].

In spite of the absence of heterogamety in zebrafish, sex-biased gene expression profiles in adult fish revealed a greater number of male-biased than female-biased genes and a higher magnitude of expression level in male-biased genes compared to female-biased genes in the gonadal tissues [17, 18]. Furthermore, a greater proportion of sex-biased genes compared to unbiased genes in zebrafish demonstrated an evidence for positive selection and therefore faster evolution of sex-biased genes [18]. In general, rapid evolution for sex-biased genes might be related to positive selection [18], sexual selection [19] or to genetic drift [20]. Zebrafish does not have much more morphological sexual dimorphism. This can be explained by the fact that accelerated evolution for sex-biased genes as a consequence of sexual selection acts simultaneously on both male- and female biased genes [18]. Nevertheless, male zebrafish show a more intense yellow colouration compared to females based on xanthophores, which is thought to be important for sexual attraction [21, 22]. Unlike mammals and birds, which have only one type of pigment cell (melanocytes), fish species have several types of chromatophores involved in the development of colour pattern (CP) [23, 24]. The interaction within and between chromatophore cell types distributed in the hypodermis of the body and the epidermis of scales and fins are required for CP formation in zebrafish [22–24]. In the larval stage, chromatophores arise directly from neural crest cells, while adult stripe patterns are developed during metamorphosis (3–6 weeks post fertilization) and display golden and blue stripes composed of yellow xanthophores, silvery or blue iridophores and black melanophores [21, 22, 25–27]. Although the anal and caudal fin stripe patterns are contiguous with the body stripes, the mechanism of fin stripe formation differs from the body stripe formation. The mechanism involved in fin CP formation in zebrafish is still largely unexplored [21, 22].

Despite the popularity of zebrafish as a well-established teleost research model animal, little information exists about the influence of elevated water temperature during embryonic development and its later effects on sex differentiation [13]. In this study, we investigate the sex ratio in response to high water temperature during embryogenesis in respect to gonadal masculinization in zebrafish. Since the regulation of sex-biased gene expression plays a major role in phenotypic dimorphism [17] and the expression of SD genes might be associated with CP genes in respect to sexual attraction [28], the investigation of underlying molecular mechanisms of SD and CP genes and their interactions can provide new insights into the genetic control of sexual dimorphism in zebrafish. Furthermore, exposure to high ambient temperatures leads to a loss of pigmentation in domesticated zebrafish [1]. Transcriptome analysis of gonads and caudal fins was performed in this study to generate profiles of the global gene expression patterns in both sexes, due to the distinct phenotypic differences in colouration between males and females in the caudal fin of zebrafish [29]. This will help fill the gap in the current knowledge regarding the association between SD and CP genes and the temperature effects on their expression in adult fish.

## Results

### Temperature effects on sex ratio

**Fig. 1:**
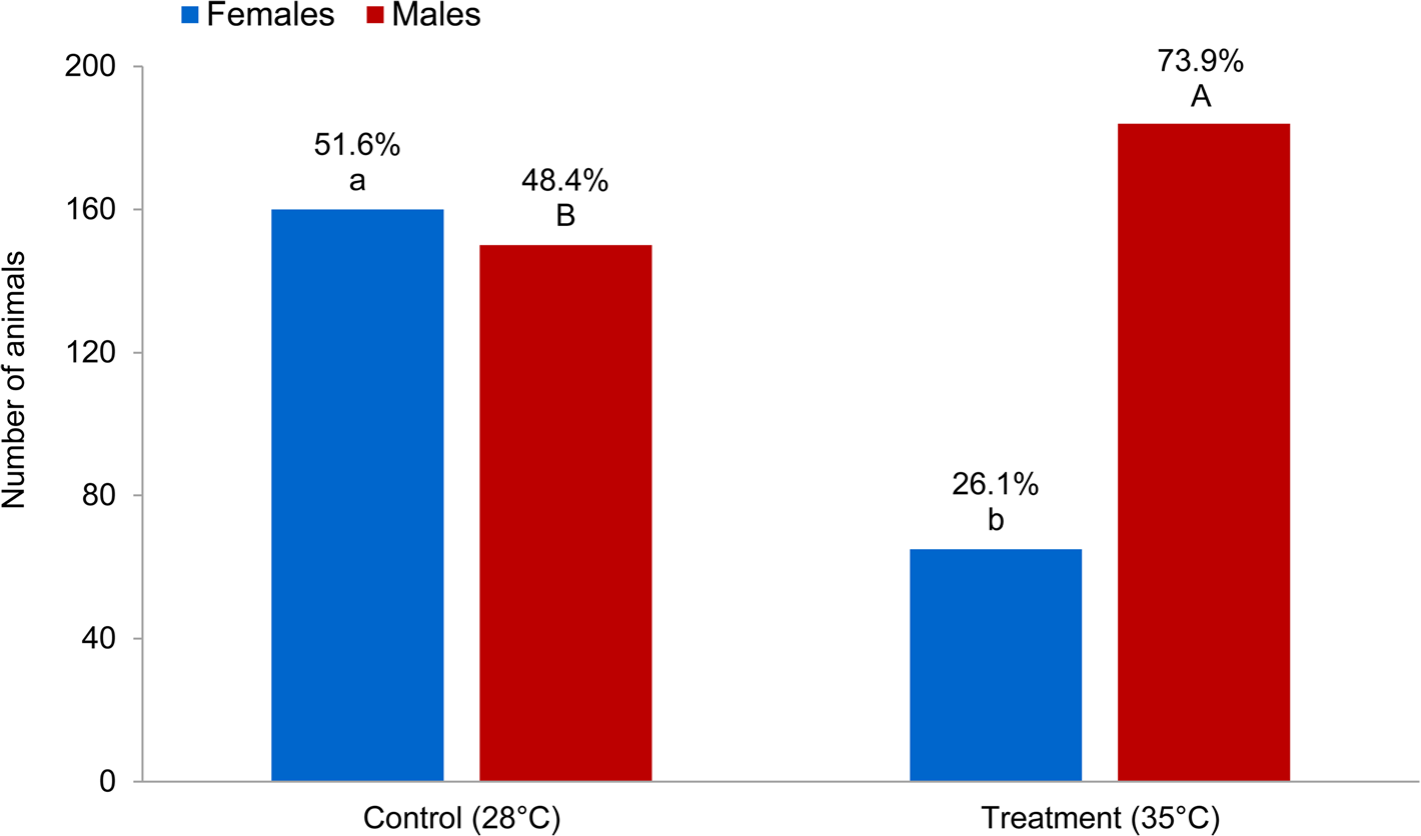
The effect of temperature treatment on sex ratio (Back-transformed least squares means in % using generalized linear model) is shown in control and temperature treatment groups. Means within treatment with different superscripts differ significantly (*P* < 0.0001). ^A-B^ Significant difference between proportion of males in control and temperature treatment, ^a-b^ significant difference between proportion of females in control and temperature treatment

Exposure of zebrafish fertilized eggs during embryonic development from 5 to 24 h post fertilization (hpf) to elevated water temperature resulted in a significantly higher male frequency compared to the control group (73.9% vs. 48.4%; Fig. 1). The ratio of females in the treated group amounted to 26.1% as compared to the control group with 51.6%. The 25.5% increase in the proportion of males under heated conditions compared to the control group suggests the induction of masculinization through the interaction between genotype and environmental factor (GxE) during SD and gonad differentiation. However, fish that do not change their sex under the influence of high temperatures are characterized as heat-resistant female animals.

### Transcriptome analysis of differential gene expression in the gonads and caudal fins

To elucidate the genetic mechanisms of association between SD and CP genes, we analysed the expression profiles of the gonad and caudal fin in treatment versus control group within males and females to find the effect of temperature treatment in different experimental groups (male treatment gonad vs. male control gonad: MTG vs. MCG; female treatment gonad vs. female control gonad: FTG vs. FCG; male treatment fin vs. male control fin: MTF vs. MCF and female treatment fin vs. female control fin: FTF vs. FCF), and males versus females within treatment and control groups to find the effect of sex in different experimental groups (male control gonad vs. female control gonad: MCG vs. FCG; male treatment gonad vs. female treatment gonad: MTG vs. FTG; male control fin vs. female control fin: MCF vs. FCF and male treatment fin vs. female treatment fin: MTF vs. FTF). A total number of 35,119 transcripts were read in RNA sequencing (RNA-Seq) expression profiles, in which the numbers of 18,871 expressed transcripts were analysed in all experimental groups. An overview of the significantly differentially expressed transcripts in comparison groups is presented in Table 1.

**Table 1:** Overview of the significantly differentially expressed transcripts in all compared experimental groups

Treatment vs. Control				Males vs. Females			
Group	Upregulated	Downregulated	Total	Group	Upregulated	Downregulated	Total
MTG vs. MCG	–	31	31	MCG vs. FCG	7705	6355	14,060
FTG vs. FCG	–	–	–	MTG vs. FTG	6483	6449	12,932
MTF vs. MCF	–	–	–	MCF vs. FCF	247	172	419
FTF vs. FCF	16	10	26	MTF vs. FTF	334	467	801

### Differentially expressed genes in temperature treatment versus control

**Fig. 2:**
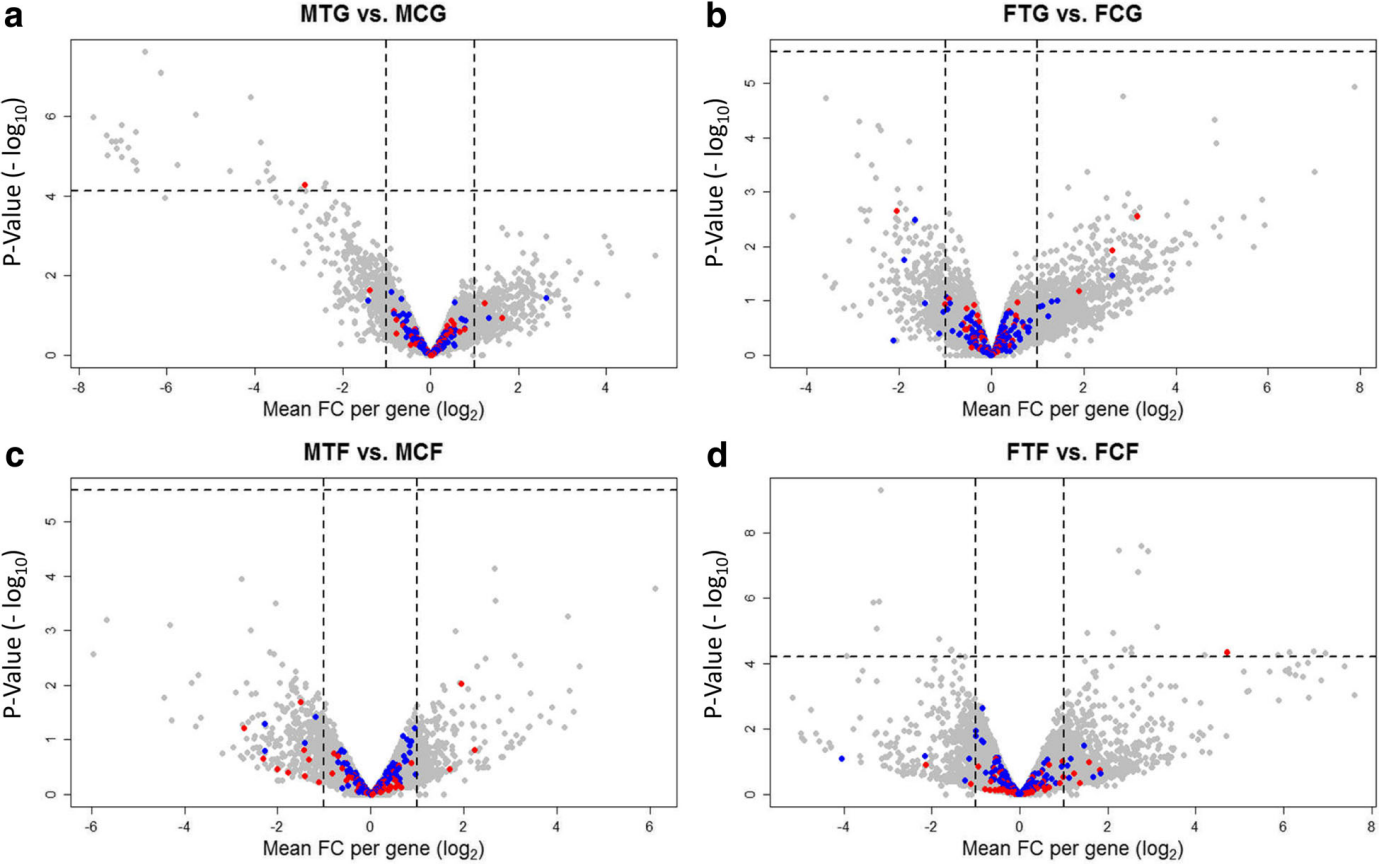
Volcano plots illustrate significantly differentially expressed genes (DEGs) in treatment versus control within males and females in the gonad and caudal fin. **a**, male treatment gonad versus male control gonad: MTG vs. MCG; **b**, female treatment gonad versus female control gonad: FTG vs. FCG; **c**, male treatment fin versus male control fin: MTF vs. MCF and **d**, female treatment fin versus female control fin: FTF vs. FCF. Each dot in the plot represents a gene with its corresponding log_2_-fold change (FC) on the x-axis and *p*-value (−log_10_) on the y-axis. Red colour dots show selected candidate sex determination genes and blue colour dots represent selected candidate colour pattern genes. The horizontal line indicates the significance threshold (false discovery rate; FDR < 0.05), while the vertical line segregates genes with log_2_FC > 1

The results of the treatment versus control comparison in male and female gonads (Fig. 2, Additional file 1) showed no significantly differentially expressed genes (DEGs) in FTG compared to FCG. However, 31 genes were down-regulated in MTG vs. MCG, where most of them play important role in kidney, liver, pancreas and gonad development (e.g. *ela2, ela2l, ela3l* and *wt1b*). This results revealed that one paralog of wilms tumor suppressor 1 (*wt1b*) (SD gene, Additional file 2; see explanation in the methods) is significantly down-regulated in MTG compared to MCG, whereas this gene is up-regulated in the FTF compared to the FCF. In general, *Wt1* encodes a zinc finger transcription factor, which is necessary for the development of different tissues including kidney, gonad, spleen and heart in fish [30]. Both paralogous of *wt1* (*wt1a* and *wt1b*) are existed in zebrafish [31, 32]. In contrast to the gonad, the transcriptome analysis in the caudal fins demonstrated no DEGs in MTF vs. MCF, while 26 significant DEGs (16 up-regulated and 10 down-regulated) were observed in FTF compared to FCF. The most significant down-regulated genes in FTF are osteocalcin genes, which are involved in mineralization of caudal fin rays and fin skeleton formation (e.g. *bglapl, f13a1* and *plod1a*) in zebrafish [33, 34]. The upregulation of the myosin heavy chain isoforms gene (*myhc4*), which plays a role in muscle cells [35], is identified in FTF vs. FCF. We also found upregulation of a small heat shock protein (*hspb11*) in FTF compared to FCF, indicating the physiological response of animals to the high ambient temperature in treatment compared to the control group. This gene is expressed during development and its expression level promotes resistance to environmental stressors. Thirteen small heat shock proteins (sHSPs) are identified in zebrafish, in which most of them are reported to be up-regulated during development under environmental heat shock [36].

### Differentially expressed genes in male versus female gonads

**Fig. 3:**
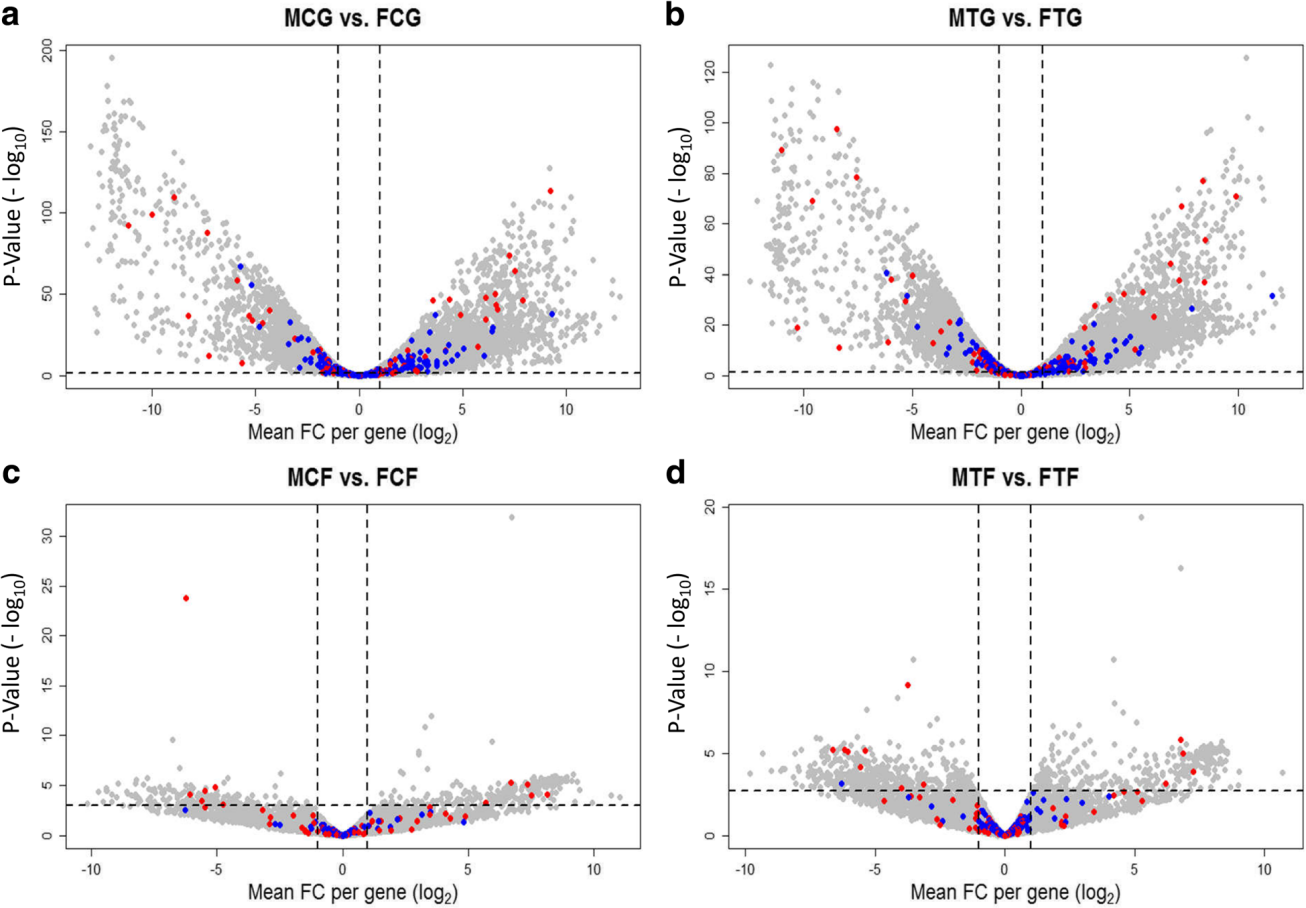
Volcano plots illustrate the result of significantly differentially expressed genes (DEGs) in males versus females within treatment and control groups in the gonad and caudal fin. **a**, male control gonad versus female control gonad: MCG vs. FCG; **b**, male treatment gonad versus female treatment gonad: MTG vs. FTG; **c**, male control fin versus female control fin: MCF vs. FCF; **d,** male treatment fin versus female treatment fin: MTF vs. FTF. Each dot in the plot represents a gene with its corresponding log_2_-fold change (FC) on the x-axis and *p*-value (−log_10_) on the y-axis. Red colour dots show selected candidate sex determination genes and blue colour dots represent selected candidate colour pattern genes. The horizontal line indicates the significance threshold (false discovery rate; FDR < 0.05), whereas the vertical line segregates genes with log_2_FC > 1

**Fig. 4:**
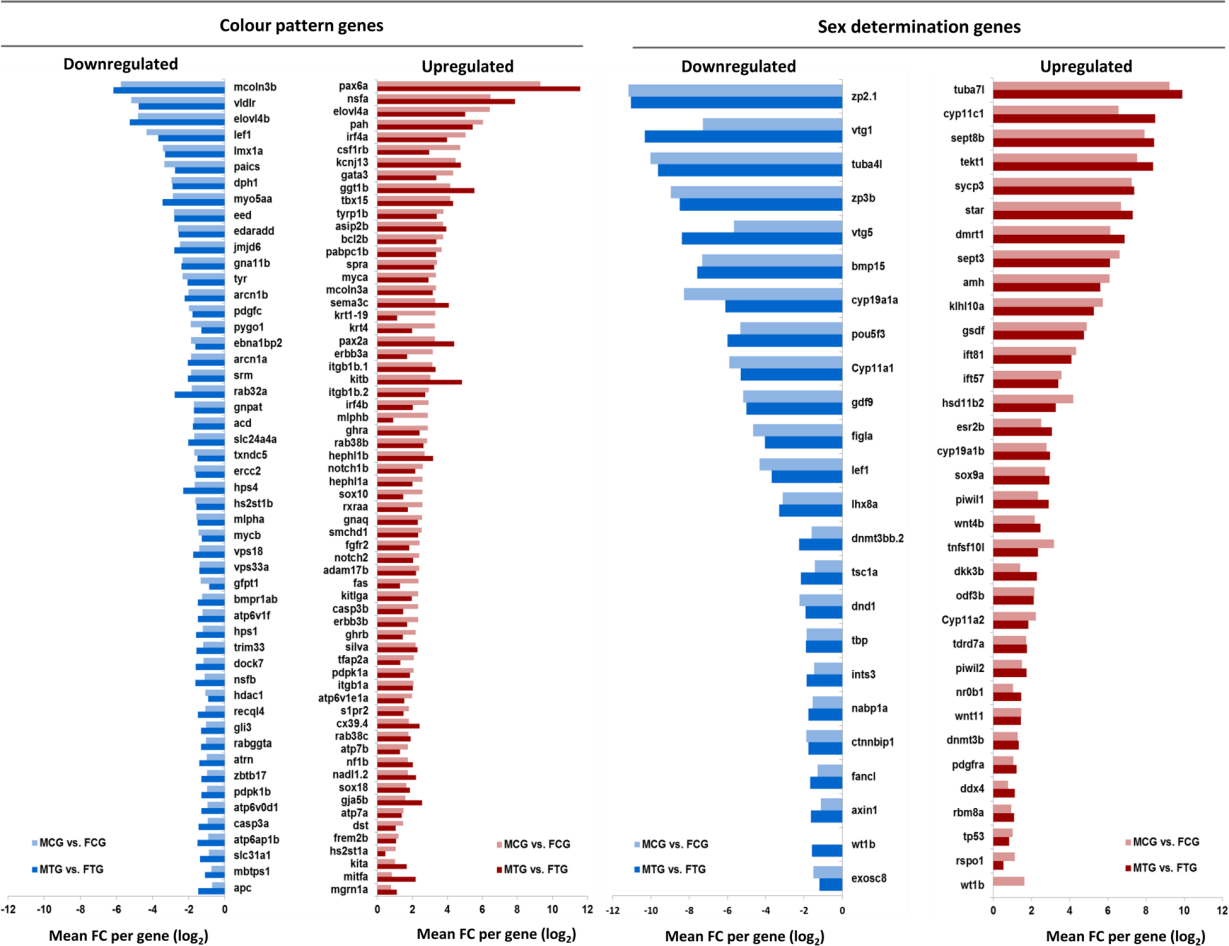
Bar charts illustrate up- and downregulation of selected candidate genes for sex determination and colour pattern in male versus female gonads within treated and non-treated groups: male control gonad (MCG), female control gonad (FCG), male treatment gonad (MTG), and female treatment gonad (FTG). The x-axis represent log_2_-fold change (FC > 1) of expressed genes, which has been shown in the threshold value of false discovery rate (FDR < 0.01)

The result of male versus female comparison in treated and non-treated groups in the gonad and caudal fin resulted in a considerable number of DEGs, illustrating the effect of sex on transcriptome profiles within treatment groups (Fig. 3, Additional file 1). The differential expression level of candidate SD and CP genes (Additional file 2; see explanation in the methods) in the gonad and caudal fin are illustrated in Fig. 3. A set of significantly DEGs from both gene groups in the gonads is presented in Fig. 4. As expected, a substantial number of up-regulated male-biased SD genes (e.g. *dmrt1, amh, gsdf*, *tuba7l* and *sox9a*) were identified in MCG vs. FCG and MTG vs. FTG (Fig. 4). The result showed that the male-biased genes related to steroidogenesis (*cyp11c1*, *esr2b*, *hsd11b2*, *star* and *cyp11a2*) and spermatogenesis (*klhl10a, odf3b* and *tekt1*) are highly up-regulated in our transcriptome profiles in treated and non-treated groups. The p53 signalling pathway-involved genes (*tp53* and *dkk3b*) responsible for testicular differentiation and a TNF-related apoptosis gene (*tnfsf10l*) are up-regulated in male gonad in both temperature treated and control groups. A greater magnitude of septin signaling transcripts (*sept3* and *sept8b*) encoding sperm tail proteins was identified in the testis. A similar high expression mode was observed for the spermatocyte development gene (*cycp3*). We also detected an upregulation of leydig cell (*pdgfra*) and sertoli cell differentiation (*sox9a*) genes in the testis.

In contrast to the upregulation of male-biased genes, down-regulated expression patterns of female-biased SD genes (e.g. *cyp19a1a, figla, gdf9* and *Bmp15*) were observed in MCG vs. FCG and MTG vs. FTG (Fig. 4). Our transcriptome analysis demonstrated that the most important folliculogenesis (*bmp15, figla, gdf9* and *lhx8a*), vitellogenesis (*vtg1* and *vtg5*) and zona pellucida proteins genes for oogenesis (*zp2.1* and *zp3b*) are highly expressed in the ovary. In addition, steroid hormone and prostaglandin signalling genes (*cyp11a1* and *cyp19a1a*) and Wnt signalling pathway genes (*ctnnbip1, lef1* and *axin1*) in the ovary were also observed to be up-regulated as compared to the testis in this study. Contrary to expectations, we observed a set of significant differentially expressed CP genes in the gonad (MCG vs. FCG and MTG vs. FTG, Fig. 4), even though these were not differentially expressed in the caudal fin, which could be due to their multifunctional character in different tissues.

### Differentially expressed genes in male versus female fins

**Fig. 5:**
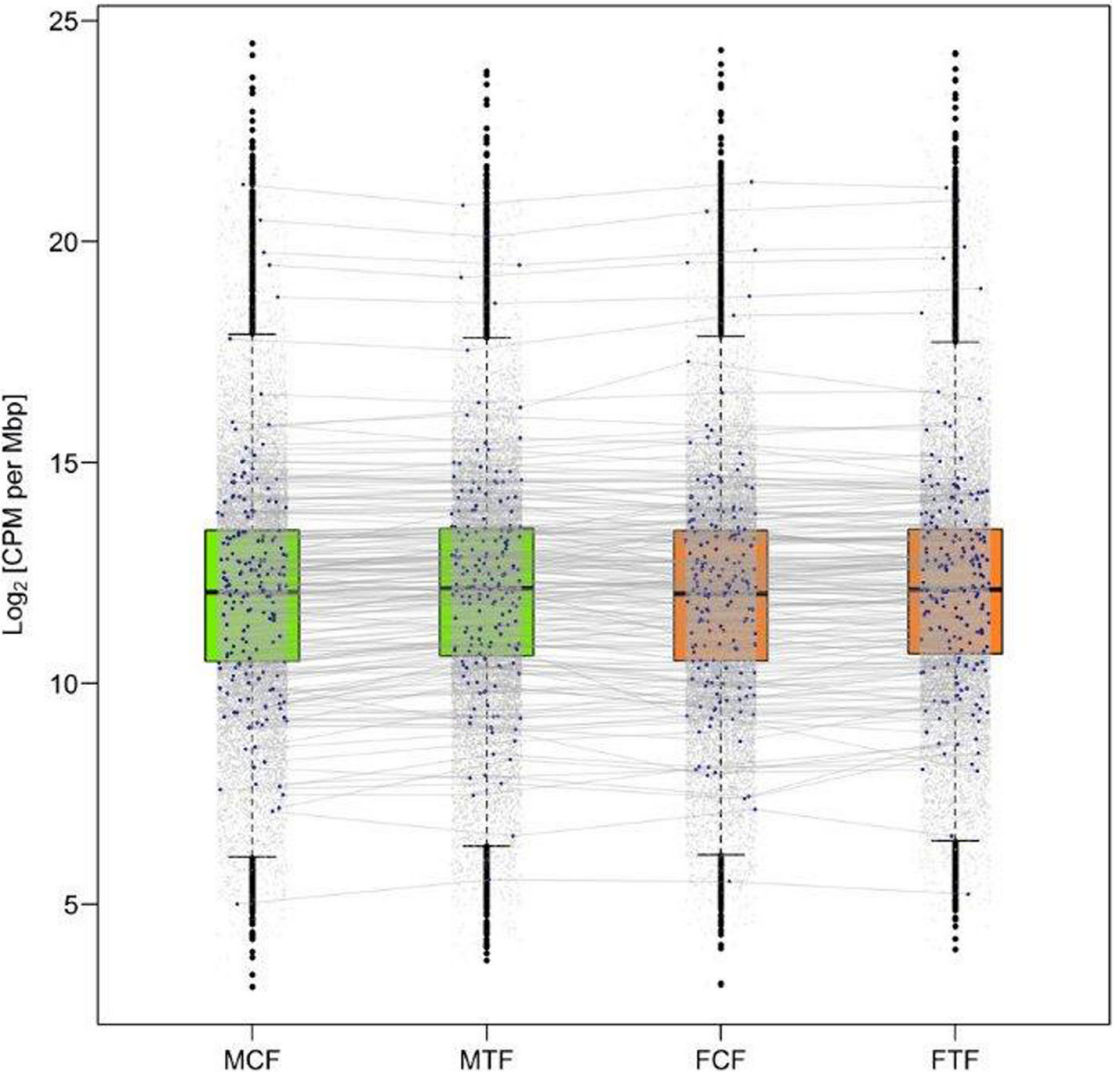
Box plots illustrate the expression of selected candidate colour pattern genes (CP) in the caudal fin. Each dot in the plot represents level of expressed gene in transcriptome profiles of four experimental groups. Blue dots show the expression level of selected candidate CP genes in male control fin (MCF), male treatment fin (MTF), female control fin (FCF), and female treatment fin (FTF), which are not significantly differentially expressed. Each blue dot in different experimental groups stands approximately on the same expression level and the expression level of corresponding dot in different experimental group connected using gray lines. The y-axis is the logarithm of counts per 1 million reads per mega base pairs (log_2_CPM per Mbp)

In contrast to the gonad, the CP genes in the caudal fin were not significantly differentially expressed in males versus females in treated and non-treated groups (Fig. 3), while a high expression magnitude of some CP genes in different groups (MCF, MTF, FCF and FTF) was observed belonging to higher than the 75 quantile of CPM (counts per million reads) distribution (Log_2_CPM per mega base pairs; Mb, 24% of selected candidate CP genes in the transcriptome profile; Fig. 5, Additional file 2). Certain candidate CP genes, such as *rsp20*, *krt4*, *rps19*, *rpl24* and *pabpc1a* were expressed at the highest level in all aforementioned groups (higher than 99 percentile; Log_2_CPM per Mb ~ 18.5). The selected candidate CP genes in this study (Additional file 2) expressed in the caudal fin were classified based on their physiological function consisting of: (1) melanophore development, e.g. *itgb1a, hdac1, mitfa, kitlga* and *kita*; (2) components of melanosomes, e.g. *silva, tyr, slc24a4* and *tyrp1b;* (3) melanosome construction, e.g. *vps18, nsfa, hps1* and *vps33a*; (4) melanosome transport, e.g. *mlpha* and *myo5aa;* (5) regulation of melanogenesis, e.g. *asip2b* and *mgrn1a*; (6) systemic effects, e.g. *elovl4b*, *vldlr, casp3a, atp7b* and *atp6ap1*; (7) xanthophore development, e.g. *csf1ra, ghrb* and *sox10*; (8) Iridophore development, e.g. *ltk, hdac1, tjp1a, tjp1b* and *vps18;* (9) pteridine synthesis, e.g. *paics, frem2b* and *spra,* in carotenoid-based colour patches; and (10) Eumelanin and Pheomelanin, e.g. *sox18, nadl1.2* and *edaradd*.

### Gene set enrichment and pathway analysis of differentially expressed genes

**Fig. 6:**
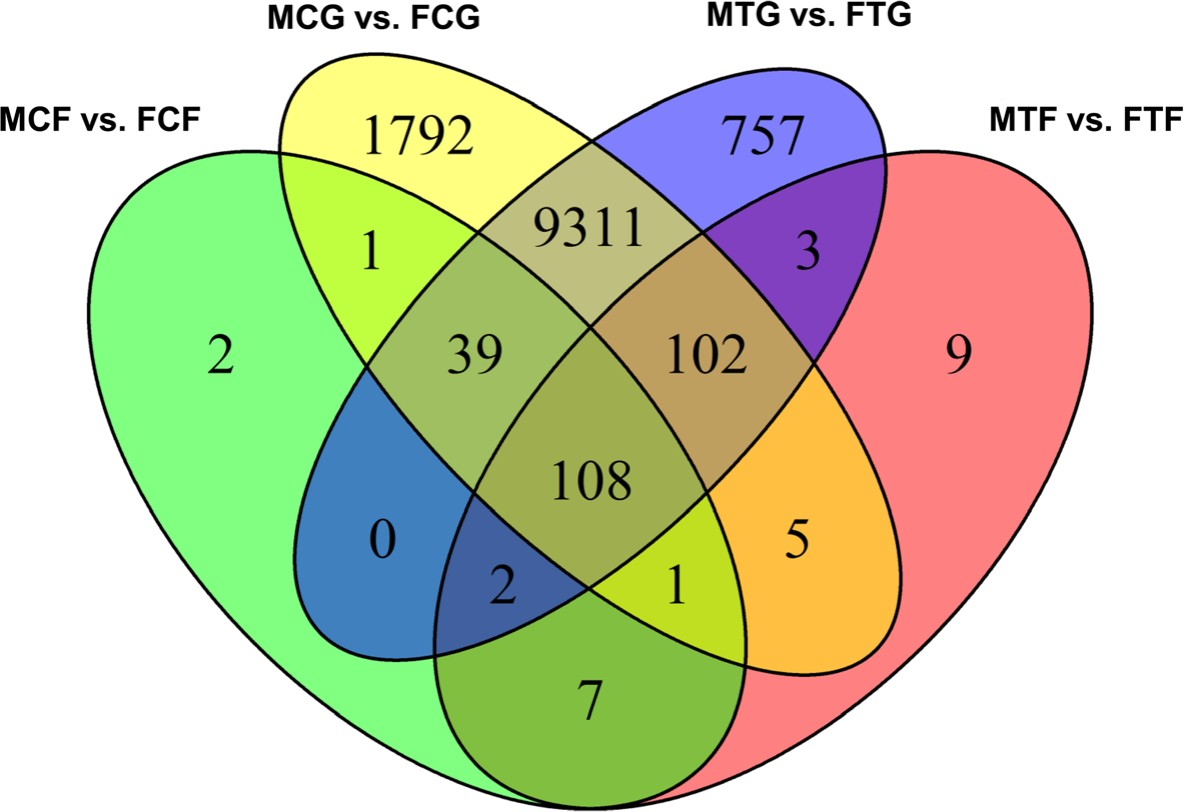
Venn diagram shows overlapping of significantly differentially expressed genes (DEGs) (false discovery rate; FDR < 0.005) using multiple comparisons with fold-changes (FC > 2): male control gonad versus female control gonad (MCG vs. FCG, yellow), male treatment gonad versus female treatment gonad (MTG vs. FTG, blue), male control fin versus female control fin (MCF vs. FCF, green), and male treatment fin versus female treatment fin (MTF vs. FTF, red)

**Fig. 7:**
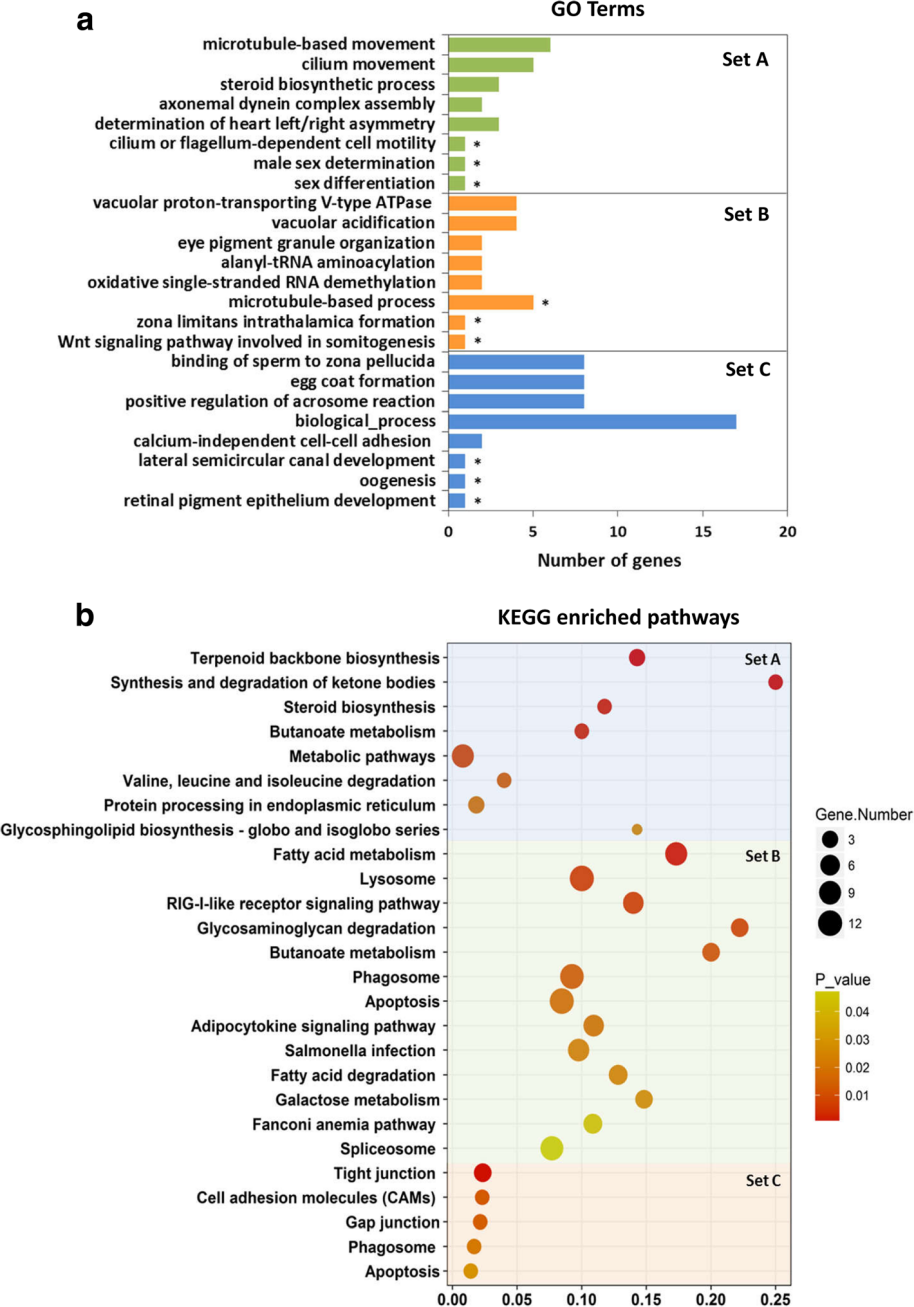
Results of gene set enrichment analysis (GSEA): **a**, Bar charts represent classification of significantly differentially expressed genes (DEGs) in gene ontology (GO) database. Categories are indicated using the number of genes present in each category and the number of genes identified in the GO library (*p* < 0.05). The vertical axis represents 5 top ranks GOs with the smallest *p*-values and in addition 3 (*) interesting GOs regarding our objectives in this study, and the horizontal axis represents the number of significantly DEGs in each GO term. **b**, Scatter plots illustrate enriched KEGG database pathways. The vertical axis represents the enriched pathway categories and the horizontal axis represents the rich factor of the enriched pathways. The size and colour of dots represent the gene number and the range of p-values, respectively. Rich factor is the ratio of differentially expressed gene number enriched in the pathway to the total gene number in a certain pathway. Set A: the enriched pathways or GOs considering significantly DEGs in MTF vs. FTF (119 genes), Set B: the enriched pathways or GOs considering significantly DEGs in MTG vs. FTG (762 genes), Set C: the enriched pathways or GOs considering significantly DEGs in overlapping between four comparison groups (108 genes)

A Venn diagram in Fig. 6 displays the overlapping associations between significantly DEGs of comparison experimental groups (MCG vs. FCG, MTG vs. FTG, MCF vs. FCF and MTF vs. FTF). In total, 762 significantly DEGs were identified in MTG vs. FTG and 119 in MTF vs. FTF (FDR < 0.005, log_2_FC > 2), which were not overlapping with their corresponding control groups, MCG vs. FCG and MCF vs. FCF, respectively. In the overlapping sets, 108 significantly DEGs (FDR < 0.005, log_2_FC > 2) were detected between four comparison groups, considering the genes involved in the gonads and caudal fins. The lists of these genes were used for Gene Ontology (GO)-enrichment in the biological process category and pathway analysis (Additional file 3). The result demonstrated that the enriched GOs in the caudal fin have functional roles in the cilia and flagella structure in the surface of the cells such as cilium movement (GO:0003341), motile cilium assembly (GO:0044458) and cilium-dependent cell motility (GO:0060285). Their corresponding enriched pathways play a role in isoprenoid biosynthesis to chondrocytes and cartilage tissues development, such as terpenoid backbone biosynthesis, synthesis and degradation of ketone bodies, and valine leucine and isoleucine degradation (Fig. 7a Set A, 7b Set A, Additional file 3). The Wnt signalling pathway involved in somitogenesis (GO:0090244), zona limitans intrathalamica formation (GO:0022006), microtubule-based process (GO:0007017) and eye pigment granule organization (GO:0008057), and the relevant pathways in ovarian and testis development namely Fanconi anemia and apoptosis were enriched in the gonad (Fig. 7a Set B, b Set B, Additional file 3).

More importantly, the result of four comparison groups illustrated the enriched GO terms related to SD and CP, such as binding of sperm to zona pellucida (GO:0007339), positive regulation of acrosome reaction (GO:2000344), egg coat formation (GO:0035803) and oogenesis (GO:0048477) for SD and lateral semicircular canal development (GO:0060875) and retinal pigment epithelium development (GO:0003406) for CP formation, in respect to biological processes.

According to pathway analysis in this group, tight junction was the top significant pathway (FDR ≤ 0.002) including important differentially expressed SD genes, *tuba4* [37] and *tuba7l* [38], which are functionally involved in ovarian follicle and in testis development, respectively. The same SD genes (*tuba4l* and *tuba7l*) were also observed in the enriched gap junction and apoptosis pathways. The genes in the tight junction pathway with a CP function in melanophore development and migration (*itgb1a and itgb1b.2*) and iridophore migration or shape change (*tjp1a* and *tjp1b*) [39] are highly expressed in our transcriptome profiles of the caudal fin. Likewise, other CP genes (*pdgfc, gna11a,* and *map 2 k1*) with a similar function in melanophore development in the gap junction pathway and the genes with systemic effects (*casp3a, casp3b* and *pdpk1b*) in the apoptosis pathway were also expressed in the caudal fin in this study (Fig. 7a Set C, b Set C, Additional file 3). Therefore, the gene set enrichment analysis (GSEA) in this study exhibited the enriched pathway genes play a role in the SD and CP and may have a co-regulation mechanism resulting in co-expression of those genes as expected.

## Discussion

### Sex ratio in response to high temperature

In this study, high water temperature treatment during embryogenesis in zebrafish resulted in masculinization, while in the control group no significant difference was found between male and female proportion. In a comparable study, the effect of elevated water temperature (at 35 °C during embryogenesis) from a mating of mitotic gynogenic males with normal females was investigated in zebrafish [13]. A mitotic gynogenetic male is developed by applying a shock treatment during the first embryonic cell cycle of a maternal haploid cell that induces two sets of chromosome in the same nucleus and forms a diploid cell. In female-heterogametic SD system, the gynogenesis process will result in an equal number of ZZ males and WW females, assuming that WW individuals are viable. However, if the W chromosome is lethal in the homozygous condition, all offspring will be male [40]. In F1-generation of crossing between the mitotic gynogenetic males and normal females the expected proportion of females would be 50% (in the case of using ZZ as a father) or 100% (in the case of using sex-reversed WW male as a father) [13]. However, the result of the aforementioned study [13] revealed a high male frequency in temperature treatment (47.5%) compared to the control group (22%), which indicates the influence of the high ambient temperature on masculinization. A recent study of heat-induced masculinization in domesticated AB strain of zebrafish showed that elevated water temperature (36 °C) during larval stage leads to a wide variety of inter-family masculinization up to 90% [1]. The results of the effect of the increase in water temperature on sex ratio in previous studies [1, 13, 14] are in agreement with the outcome of this study. In many other fish species, which have a similar sexual plasticity, the effect of elevated water temperature on masculinization has been demonstrated [41–43]. Therefore, our result emphasizes the interplay between genetic and environmental influences on SD in zebrafish and confirms earlier studies on polygenic SD (PSD) in domesticated strains [2, 3, 44] during embryonic [13, 14] and larval stages [1]. However, little is known about the molecular basis of the effects of high water temperature during embryogenesis on SD in zebrafish and many closely related species.

### Transcriptome profiles of differentially expressed genes in different temperature groups

Transcriptome analysis of gonads and caudal fins were performed to investigate the underlying genetic mechanism of the association between SD and CP genes and the effect of high ambient temperature on their expression profiles in zebrafish. Gene expression analysis in treatment versus control group within males and females is showed a set of DEGs in MTG vs. MCG and in MTF vs. MCF. One of these genes, *Wt1,* is down-regulated in MTG vs. MCG, which is related to the development of different organs in zebrafish. Most studies on the function of *wt1* in zebrafish have been focused on the embryonic kidney, the pronephros, development [31, 32]. However, *Wt1* is necessary for urogenital ridge development and identifying the adrenal-gonadal primordium (AGP), and both paralogous are seems to be required for kidney and gonad development in zebrafish [45]. These paralogous have a clear role in pronephric glomerular formation of kidney during embryonic development in zebrafish. *Wt1a* is expressed at the early of pronephros development to form glomerular structures, while *wt1b* is expressed in the later stage of nephrogenesis [32]. In adult zebrafish, a high expression level of both *wt1* genes can be found in the kidney, gonad, heart, spleen, and muscle tissues [30, 31]. Expression of *wt1* at AGP is essential for the steroidogenic interrenal cells development in cooperation with *ff1b* (*nr5a1a*) gene (the equivalent of mammalian *SF-1*), suggesting its important role during development of gonadal primordium in zebrafish [45–47]. In a recent study, expression of *wt1a* gene was observed in the zebrafish testis and is identified as a pro-male gene [48]. In medaka, *wt1a* is expressed in the somatic cells of the gonadal primordium, but *wt1b* is expressed in the later stage of development. The somatic gonadal cells in medaka arise from the lateral plate mesoderm (LPM) at the early of embryonic development [49]. *Wt1a* is expressed in the LPM during early embryogenesis and later in the somatic cells of the primordial gonad. *Wt1b* coordinates with the *wt1a* to develop both pronephros and gonad developments. Expression of both genes in the medaka displays a strong effect on the maintenance and survival of the number of PGCs during gonad development [50]. Our transcriptome profiles revealed that thermal treatment during embryogenesis might influence the expression mechanism of the *wt1b* gene in the zebrafish during gonad differentiation, which in turn may result in masculinization. Since the number and survival of PGCs are necessary for ovarian development in zebrafish [10, 11], we hypothesized that downregulation of *wt1b* in MTG compared to MCG during embryogenesis in our study may cause the reduction in the number of PGCs, leading to masculinization in heat-treated animals. However, its underlying molecular mechanism requires further research. Furthermore, the high expression of *hspb11* in FTF vs. FCF with regard to the thermal stress has been observed in this study. Generally, sHSPs act as molecular chaperones to prevent the aggregation of denatured proteins or to reverse improper protein associations during cellular stress [51–53]. This function is an important physiological role of sHSPs, which implicitly induces of the expression of sHSPs by a variety of stressors [52, 53]. In zebrafish, the high expression of *hspb11*, as a member of sHSPs family, was observed in somites, heart, dorsal mid- and hindbrains after heat shock [36, 51]. In this study, we observed a high expression of *hspb11* in the caudal fin of zebrafish in response to the elevated water temperature.

### Transcriptome profiles of differentially expressed genes in different sexes

Within a substantial number of significantly differently expressed sex-biased genes, a series of pro-male and pro-female genes were identified within two temperature groups. One of the most important pro-male genes is *dmrt1*, which has a sex-specific role in testis development and its expression is necessary for the transcriptional regulation of *amh*. *Amh* is a key testis gene and normally expressed in the sertoli cells after testicular differentiation and inhibits the ovarian aromatase gene expression (*cyp19a1a*), resulting in gonadal masculinization in zebrafish. *Amh* is downstream of *dmrt1* in zebrafish and its expression is regulated by *dmrt1* in somatic cells of testis. These suggest that *dmrt1* plays a male-specific role in zebrafish and loss or decrease of its expression interferes with normal male sexual development [7, 48, 54]. A similar expression pattern of *dmrt1* in other fish species such as medaka [55], tilapia [56], European seabass [41] and pejerrey fish [57] has been observed in temperature-induced masculinization. Furthermore, steroidogenic enzymes, encoded by *cyp11c1* and *hsd11b2* pro-male genes [48], are required for 11-oxygenated androgen production, which are up-regulated in testis in this study. In contrast to the pro-male genes, *Cyp19a1a* is a key regulator gene in ovarian development (pro-female gene) encodes the P450 aromatase enzyme, responsible for conversion of androgens to estrogens in the female gonad, and is a downstream gene of *Bmp15*. Oocyte-produced signalling protein, *Bmp15,* is necessary for expression of *cyp19a1a* and maintains of adult female sex differentiation in zebrafish. Hence, loss or downregulation of the expression of *Bmp15* leads to a reduction in the expression of *Cyp19a1a* and consequently to a disruption of ovarian development [58]. Additionally, the expression of zp family member genes (*zp2* and *zp3*), which are up-regulated in the ovary, encodes the major protein components of zebrafish egg chorion (glycoprotein layer) and is active in the development of oocytes [59]. Finally, a high number of significantly sexually dimorphic transcripts are identified in our study (Table 1), in which some of them were already identified in other studies as pro-male and pro-female genes [1, 48] in accordance with our results. During the zebrafish SD and gonad differentiation, the actions of several pathways regulate the sexual fate of an organism to develop either a testis or an ovary [60]. GSEA in this study revealed that Fanconi anemia and apoptosis pathways with SD function in the ovary and testis respectively were enriched in the comparative analysis of adult gonads, supporting the observation of previous studies [1, 61].

Surprisingly, similar to the sex-biased gene expression in the gonads, a significant set of CP genes was differentially expressed in the gonads. Many of the CP genes involved in pigment cell development have other functions not related to the pigmentation and they are considered as duplicated genes that have arisen from ancestral genome duplication specific to ray-finned fish [62]. A study on retroduplication in mammals and *Drosophila* demonstrated that “testis” has a central role in fixation and functional evolution of new genes. Indeed, testis is an evolutionary tissue with the most rapidly evolving organ, which may represent the target tissue for the evolution of new genes [63, 64]. These genes and their functions could become adopted into other tissues over time [64, 65]. These novel genes can impact the evolution of cellular, physiological, morphological, behavioral and reproductive phenotypic traits [64]. The neofunctionalisation of one copy of many duplicated genes implicated a secondary character, but is interestingly co-opted to a primary role after duplication [66]. The *kit* system is a well-studied duplicated gene family known to play an essential role in pigmentation and ovarian development. *Kitlga* and *kita* genes are more specialized for melanophore development and migration [67, 68]. *Kit* ligand and their receptor also play an important role in spermatogenesis and oogenesis in adult zebrafish [69, 70]. *Kitlga* is expressed in the trunk of the body in zebrafish during melanocyte migration stage and later in the skin, and its receptor (*kita)* is required for melanocyte survival [67]. *Kitlga* and *kita* genes are also expressed in the ovarian somatic follicle cells and are responsible for oocyte maturation. Since the target tissue of endocrine hormones for regulation of folliculogenesis is somatic follicle cells, the expression of *Kitlga* in somatic cells possesses a function as an external stimulating factor on IGF-I mediator in PI3K-Akt pathway in the gonad [70]. *Kit* system genes in mouse testis play an important role in signalling cascades initiated by *Kit* in PGCs, spermatogenesis and oogenesis. In mouse testis, *kit* ligand and its receptor is expressed in sertoli cells during spermatogonial development and in leydig cells, which have considerable influence on the endocrine function in mouse spermatogenesis [71]. Interestingly, in this study we found the upregulation of *kit* genes in the male gonads compared to female gonads, which emphasizes the importance of the *kit* system genes in the spermatogenesis process in zebrafish. To the best of our knowledge, we are the first to report the expression of a series of CP genes in the zebrafish reproduction system, but its underlying biological reason is still unknown and deserves further research.

Contrary to the gonads, no significantly differentially expressed CP genes were observed in the caudal fin. However, some of those genes showed a high level of expression in both temperature groups. For CP formation in zebrafish, chromatophores arise directly from neural crest cells during embryonic development and later during metamorphosis from stem cells to generate the adult pigment pattern through interactions between different chromatophore cell types [21, 22, 72, 73]. Besides this cell-cell interaction mechanism, agouti-signalling peptide (ASIP) has been observed to control the dorso-ventral patterning in the skin of adult zebrafish (60 and 210 days post fertilization: dpf), resulting in the graded expression of melanin synthesis-involved genes such as *mitfa, tyrp1b* and *dct.* However, ASIP was not detected to contribute to the pigmentation of the adult fins [74] due to different mechanisms of stripe formation in the body and fins [21, 22]. Nevertheless, the studies of caudal fin regeneration have been shown that the regeneration cells are able to remember their former locations and patterned the caudal fin tissue after injury in adult zebrafish. The expression activity of the *kitlga* gene is demonstrated to promote the recovery of melanocytes during the regeneration of adult zebrafish caudal fin [75–77]. Sex-biased gene study in the tail of guppy has revealed that several male-biased genes encoded proteins with pigment biosynthesis functions (e.g. *kita, kitb, mitfa, mitfb, tyrp1b, dct* and *xdh*) [28]. In adult zebrafish, sexual dimorphism is illustrated by a brighter yellow colouration in males than in females [22]. Therefore, assuming that the differential expression of CP genes may be important for sex-dependent colour patterning during early stages of development, in adults; at least their expression levels are more constant. The observed differences in colouration between males and females could be due to post-transcriptional regulation of key enzymes involved in pigment synthesis and distribution. In GSEA, the tight junction and gap junction pathways has been enriched in this study, in which the genes involved in these pathways have a function in CP development in zebrafish, as their expression was observed in our transcriptome profiles. Interestingly, *tuba4* and *tuba7l*, which play a role in SD in zebrafish, were differentially expressed in these pathways, but their role in CP of zebrafish needs further investigation. We also observed a few SD genes (e.g. *cyp19a1b, zp3b, tekt1, dmrt1* and *sycp3*) are differentially expressed in the MCF vs. FCF and MTF vs. FTF. Taking into account the fact that the correlations between the expression level of these genes in the gonads and in the caudal fins are relatively weak, the genetic cause of these gene expressions requires further research.

## Conclusions

Elevated water temperature during embryogenesis resulted in male-biased sex ratio in this studied zebrafish population, which supports the hypothesis of a PSD system in domesticated strains. In this study, transcriptome analysis of gonads revealed the activation of pro-male gene expression and repression of pro-female gene expression in male compared to female gonads, leading to gonadal masculinization in laboratory zebrafish. However, unexpected differential expression patterns of the most CP genes were observed in the gonad, suggesting the neofunctionalisation of those genes in zebrafish reproduction system. Contrary to the gonad, the different colouration in the caudal fin of adult fish was not due to the differential expression of CP genes, even though a high expression magnitude of those genes was observed in both sexes. The observed differences in colouration between males and females may be due to a post-transcriptional regulation of key enzymes involved in pigment synthesis and distribution. Furthermore, we identified a subset of enriched pathways (tight junction, gap junction and apoptosis) containing both SD and CP genes, which may play a pivotal role in regulation of phenotypic sexual dimorphism, regarding the differences in CP of two sexes in adult zebrafish.

## Methods

### Fish stocks and husbandry

The Singapore strain of zebrafish was used in this study. This strain was directly imported from a breeding farm in Singapore in 1990 by the Company Aquafarm Ryba Zeven, GmBH (Zeven, Germany) [78, 79] and kept in the aquaculture facilities of the University of Goettingen for research purposes in accordance with approved institutional guidelines. The zebrafish population was kept in mixed sex groups at 28 ± 0.5 °C. The photoperiod regime was applied 12-h light/12-h dark per day. The fish were fed two times a day with commercial food (Tetramine junior, Germany) and freshly hatched *Artemia salina* nauplii.

### Temperature treatments

In this study, we used the fertilized eggs derived from full-sib family in equal proportions in order to investigate the effect of high water temperature on sexual plasticity and sex related colour patterning. For this proposed, two temperature treatments were designed: 1) in the first treatment, the animals were kept at the constant temperature of 28 °C throughout the experiment (control group), and 2) in the second treatment, the eggs were exposed to the high water temperature of 35 °C [16, 80–82] during embryogenesis from 5 to 24 hpf (treatment group). This development time is a critical phase of embryonic development known as segmentation stage (between gastrula to pharyngula period) [13, 14, 83]. After treatment, the heat-exposed groups were returned to the control temperature at 28 °C. All experimental groups were then kept under the same environmental conditions until sexual maturity. In order to avoid the heat stress, the temperature of treatment group was gradually increased and/or decreased to the target temperature in this study. To ensure the accuracy of the experiment, the temperature of the experimental groups was controlled daily throughout the study. Both temperature treatment and control groups were mixed separately in 36-l tanks (AquaBox® by Aqua Schwarz GmbH, Goettingen, Germany) 2 weeks after the eggs had hatched until sexual maturity to eliminate the effects of population density within tanks. After sexual maturity, the urogenital papilla was examined and, in the unclear case, the microscopic examination was performed to determine the sex of each individual. In this study, the sex of a total number of 559 individuals was determined in all experimental groups. All husbandry facilities, fish management and water quality control, animal care and feeding are described by details in Hosseini et al. [14].

### Tissue sample collection

**Fig. 8:**
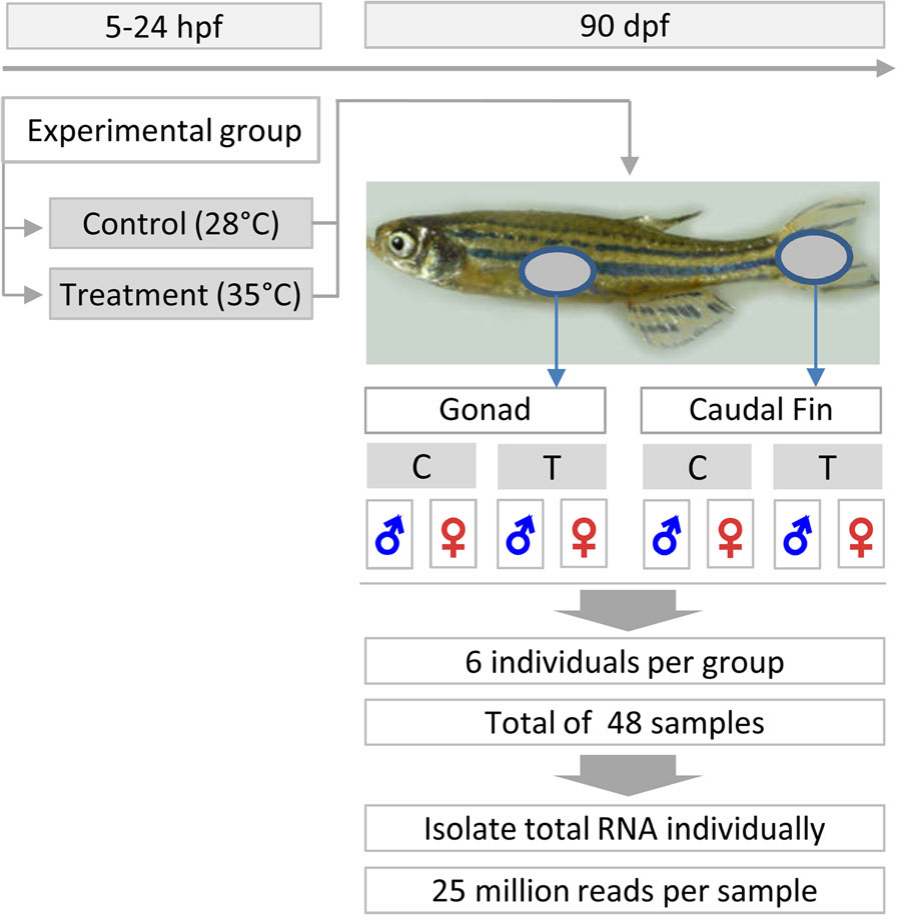
General overview of experimental design and tissue sample (Gonad and Caudal fin) collection for RNA_Seq analysis in this study. Total RNA was isolated from adult zebrafish gonad and caudal fin tissues of each individual separately and used for transcriptome analysis. Hpf: hours post fertilization; dpf: days post fertilization; C: control; T: temperature treatment

Since the distinct phenotypic sexual dimorphism in colouration between males and females was observed in the caudal fin, the tissue samples of caudal fins and gonads of adult zebrafish were collected for transcriptome analysis. Tissue samples of 6 individuals in each experimental group (control male, control female, treatment male, and treatment female) from two different tissues (gonad and caudal fin) were collected after sexual maturity. A total number of 48 tissue samples (24 caudal fin and 24 gonad samples) were used for transcriptomic analysis (Fig. 8). For this purpose, the animals were sacrificed and tissues were carefully dissected. Tissue samples were stored in RNA stabilization solution, RNAlater® Tissue Collection (Thermo Fisher Scientific, Germany) and kept at − 20 °C until starting the molecular laboratory genetic analysis.

### RNA extraction and sequencing

Total RNA of the gonad tissues was isolated using a RNeasy Plus Mini kit (Qiagen, Hilden, Germany) and total RNA from the caudal fin samples was extracted by a RNeasy Fibrous Tissue kit (Qiagen, Hilden, Germany) according to the manufacturer’s protocol. The RNA quantity and quality was measured using a Qubit 2.0 Fluorometer (Thermo Fisher Scientific, Waltham, USA) and RNA Screen Tape on an Agilent Bioanalyzer 2100 (Agilent, Santa Clara, USA). The RNA integrity number (RIN) of the most samples was> 7, exception of two samples with RIN scores of 6.1 and 5.6. The sample libraries preparation was performed from 500 ng input total RNA using the TruSeq stranded mRNA kit, and sequenced on an Illumina HiSeq4000 platform aiming for 25 million 2 × 75 bp paired-end reads per sample.

### RNA read alignment and gene counting

Quality assessment of raw sequencing data was conducted using FastQC (version 0.11.4) [84] and MultiQC (version 1.3) [85] for forward and reverse reads. Due to the overall high quality of the reads, no samples had to be excluded from the analysis. FASTQ files were processed with Trimmomatic (version 0.36) [86] to remove the low quality bases and Illumina adapters. The average percentage of paired reads that survived in trimming was 93.5% ± 0.5%. RNA read alignments were then mapped to the *Danio rerio* genome assembly version GRCz10 (GCA_000002035.3), which was downloaded from Ensembl [87] using STAR-2pass (version 2.4.2a) [88] and resulted in an average mapping success rate of 90.1% ± 3.3%. Finally, FeatureCounts (version 1.4.3) [89] was utilized to count the number of reads mapped for each gene with an average successful read assignment of 73.6% ± 4.9%. All aforementioned software packages were run using the standard settings.

### Differential gene expression and gene set enrichment analysis

All RNA_Seq analyses in this study were performed in the R-Statistics program [90] using the “edgeR” package [91]. For each tissue (gonad and caudal fin), we conducted four comparisons - (1) males vs. females within treatment and control groups, and (2) treatment vs. control within males and females– to assess the effect of sex and temperature treatment, respectively. This resulted in 8 comparisons, for which we performed a differential gene expression analysis (DGEA).

To investigate the significantly differentially expressed SD and CP genes, we prepared two gene lists for candidate genes: (1) a list containing genes that have been associated with SD; and (2) that have been associated with CP, which are addressed in the different literatures and NCBI gene database for zebrafish. For selection and classification of CP genes, we used coat colour categories (http://www.espcr.org/micemut) [62] and additional information given by zebrafish database (ZFIN; http://zfin.org) [92]. A total number of 79 SD and 213 CP genes were used as selected candidate genes in this study (Additional file 2).

For the DGEA, we only considered genes with more than 1 CPM to avoid unreliable results across all samples, of at least one of the eight experimental groups: male control gonad (MCG), female control gonad (FCG), male treatment gonad (MTG), female treatment gonad (FTG), male control fin (MCF), female control fin (FCF), male treatment fin (MTF), and female treatment fin (FTF) to be expressed and use them for further analyses. This resulted in a matrix of 18,871 genes for 48 samples (6 biological replicates per experimental group). The DGEA was conducted using a negative binomial model and the exact test in edgeR. In general, each DGEA was comprised of three main steps: (1) normalization of gene counts for sample-specific effects (sequencing depth and RNA composition); (2) fitting a negative binomial model to the count data to estimate relevant dispersion parameters; and (3) performing an exact test for the negative binomial distribution to compare whether gene expressions are significantly different between two conditions.

To account for multiple testing, we employed the false discovery rate (FDR) approach by Benjamini and Hochberg [93]. We then compared the results of the 8 comparisons and obtained three sets of significant DEGs (FDR < 0.005, log_2_FC > 2), for which we performed a GSEA to gain insight into their biological interpretations. To this end, we first created a pathway and Gene Ontology (GO) annotation based on the Kyoto Encyclopedia of Genes and Genomes (KEGG) database [94] and GO database [95], respectively. Then, for each pathway and GO and each set of significant genes, we performed a Fisher’s Exact test to identify pathways and GOs that are enriched with significant genes. Hereby, we considered pathways or GOs that contain at least one significant gene. Due to the small number of animals and since the Fisher’s Exact test is known to be conservative, we are aware that our test might be under powered. Therefore, we considered GOs that are highly ranked (the first 5 GOs with the smallest *p*-values) and in addition 3 other important GOs regarding our objectives in this study as potential candidates for discussion. The GOs and pathways with the p-values less than 0.05 were investigated to be enriched in DGEA. In addition, GOs and pathways with corrected p-values after multiple test correction (Benjamini–Hochberg method) were considered to be significantly enriched in the DGEA and are presented in Additional file 3.

### Statistical analysis

Since sex is a binary variable characterized by 0 and 1 values, and its consideration as the independent variable in a statistical model does not represent the assumption of a normal distribution, and a statistically appropriate function to describe this association is the logistic model, therefore, a linear logistic model was used to investigate the effect of temperature treatment on sex determination. In this case, the dependent variable (y_i_) represents the value 1 for the probability to be male (π_i_) or 0 for the probability to be female (1- π_i_) for the observation i.

The logit link function [96] is defined by $Logit\left(\frac{\pi_i}{1-{\pi}_i}\right)={\eta}_i$ where η_i_ is the probability of being male on the logit scale.

The GLIMMIX procedure of SAS version 9.3 [97] was then used to analyse the data according to the following model: *η*_*i*_ = *μ* + *α*_*i*_.where π_i_ is the probability of being male, μ is the general mean effect, α_i_ is the fixed effect of temperature treatment (i = 1: temperature-treated eggs 35 °C, i = 2: control group 28 °C). Least squares means were estimated on the logit scale and then back transformed using the inverse link function to the original scale (probability to be male) [98].

## Additional files

Additional file 1: **Table S1.** Differentially expressed genes in male treatment gonad vs. male control gonad (MTG vs. MCG)**. Table S2.** Differentially expressed genes in female treatment gonad vs. female control gonad (FTG vs. FCG)**. Table S3.** Differentially expressed genes in male treatment fin vs. male control fin (MTF vs. MCF)**. Table S4.** Differentially expressed genes in female treatment fin vs. female control fin (FTF vs. FCF)**. Table S5.** Differentially expressed genes in male control gonad vs. female control gonad (MCG vs. FCG)**. Table S6.** Differentially expressed genes in male treatment gonad vs. female treatment gonad (MTG vs. FTG)**. Table S7.** Differentially expressed genes in male control fin vs. female control fin (MCF vs. FCF)**. Table S8.** Differentially expressed genes in male treatment fin vs. female treatment fin (MTF vs. FTF). (XLSX 7713 kb)

Additional file 2: **Table S9.** Selected candidate sex determination genes used in this study. **Table S10.** Selected candidate colour patterning genes used in this study. **Table S11.** Quantile distribution of colour patterning genes in the caudal fin in the transcriptome profile (Log_2_CPM per mega base pairs). (XLSX 1847 kb)

Additional file 3: **Table S12.** List of differentially expressed genes in male treatment fin vs. female treatment fin (MTF vs. FTF), which are used for gene set enrichment analysis (GSEA). **Table S13.** List of differentially expressed genes in male treatment gonad vs. female treatment gonad (MTG vs. FTG), which are used for gene set enrichment analysis (GSEA). **Table S14.** List of differentially expressed genes in overlapping between comparison groups, which are used for gene set enrichment analysis (GSEA). **Table S15.** Enriched pathways in male treatment fin vs. female treatment fin (MTF vs. FTF). **Table S16.** Enriched pathways in male treatment gonad vs. female treatment gonad (MTG vs. FTG). **Table S17.** Enriched pathways in overlapping between comparison groups . **Table S18.** Enriched GOs in male treatment fin vs. female treatment fin (MTF vs. FTF). **Table S19.** Enriched GOs in male treatment gonad vs. female treatment gonad (MTG vs. FTG). **Table S20.** Enriched GOs in overlapping between comparison groups. (XLSX 248 kb)

## Abbreviations

AGP: Adrenal-gonadal primordium
ASIP: Agouti-signalling peptide
CP: Colour pattern
CPM: Counts per million reads
DEGs: Differentially expressed genes
DGEA: Differential gene expression analysis
dpf: Days post fertilization
FC: Fold change
FCF: Female control fin
FCG: Female control gonad
FDR: False discovery rate
FTF: Female treatment fin
FTG: Female treatment gonad
GO: Gene Ontology
GSEA: Gene set enrichment analysis
GxE: Genotype x environment interaction
hpf: Hours post fertilization
KEGG: Kyoto Encyclopedia of Genes and Genomes
Mb: Mega base pairs
MCF: Male control fin
MCG: Male control gonad
MTF: Male treatment fin
MTG: Male treatment gonad
PGCs: Primordial germ cells
PSD: Polygenic sex determination
RNA-Seq: RNA sequencing
SD: Sex determination
